# Hantavirus infections: an emerging zoonotic threat in the context of ecological change

**DOI:** 10.1016/j.nmni.2026.101763

**Published:** 2026-05-12

**Authors:** Christian Joseph N. Ong, Kevin Smith P. Cabuhat, Jamil Allen G. Fortaleza, Rich Milton R. Dulay

**Affiliations:** a Department of Biology, College of Science, De La Salle University, Manila, Philippines; b Basic Education Department, La Consolacion University Philippines, Bulacan, Malolos, Philippines; c National University Philippines, Manila, Philippines; d Center for Tropical Mushroom Research and Development, Central Luzon State University, Nueva Ecija, Philippines

Dear Editor,

Hantaviruses, belonging to the family *Hantaviridae*, are increasingly recognized as significant zoonotic pathogens responsible for severe human diseases, including hemorrhagic fever with renal syndrome (HFRS) and hantavirus cardiopulmonary syndrome (HCPS). Although traditionally considered geographically confined, recent epidemiological data indicate a gradual expansion in the incidence and geographic distribution of hantavirus infections, raising concerns regarding their re-emergence as a global public health threat [1,2]. Recent reports of suspected hantavirus exposure linked to rodent infestation aboard cruise ships further highlight the continuing public health relevance of hantaviruses in highly mobile and densely populated travel settings, where confined environments may facilitate exposure to aerosolized rodent excreta and complicate outbreak containment. Such incidents underscore the importance of environmental sanitation, rodent surveillance, and rapid risk communication in international travel and tourism sectors.

Hantavirus transmission primarily occurs through inhalation of aerosolized excreta from persistently infected rodent reservoirs, with each viral species exhibiting strong host specificity. For instance, *Hantaan virus* is associated with *Apodemus agrarius* in Asia, whereas *Sin Nombre virus* is linked to *Peromyscus maniculatus* in North America. In South America, the Andes virus represents a particularly important hantavirus species because it is one of the few hantaviruses with documented person-to-person transmission, primarily reported in Argentina and Chile. Andes virus infection is strongly associated with severe HCPS and high case fatality rates, emphasizing its epidemic potential and unique public health implications. Clinically, HFRS and HCPS differ in pathophysiology but share a hallmark of increased vascular permeability, leading to renal failure or cardiopulmonary collapse, respectively. Case fatality rates vary from <1% in milder HFRS forms to approximately 30–40% in HCPS, underscoring the virulence of certain hantavirus strains [2,3].

The re-emergence of hantavirus infections is increasingly associated with environmental and anthropogenic drivers. Climate variability, including altered precipitation and temperature patterns, significantly influences rodent population dynamics and virus transmission cycles. Notably, El Niño–associated climatic events have been linked to increased rodent abundance and subsequent hantavirus outbreaks [4]. Additionally, deforestation, agricultural expansion, and urban encroachment into wildlife habitats enhance human exposure to infected reservoirs, particularly in low- and middle-income countries where ecological disruption is most pronounced [1,5]. These ecological pressures may facilitate the emergence of novel transmission settings beyond traditional rural exposures, including occupational, recreational, and travel-associated environments.

From a diagnostic standpoint, hantavirus infections remain underrecognized due to their nonspecific prodromal symptoms, which overlap with other endemic febrile illnesses such as dengue, leptospirosis, and influenza. This diagnostic ambiguity is particularly relevant in Southeast Asia, where co-endemic zoonoses complicate clinical differentiation. Early laboratory confirmation using serological assays (IgM/IgG ELISA) and molecular techniques (RT-PCR) is essential for accurate case identification and outbreak control [2]. Moreover, enhanced genomic surveillance is increasingly important for identifying emerging hantavirus variants and monitoring transmission dynamics, especially in regions experiencing environmental change and expanding human–wildlife interaction.

In the absence of widely available targeted antiviral therapies and globally licensed vaccines, prevention remains the primary strategy for hantavirus control. Public health interventions should emphasize rodent control, environmental sanitation, and risk communication to minimize human exposure. Importantly, the integration of hantavirus surveillance into a One Health framework—linking human, animal, and environmental health systems—offers a strategic approach for early detection and mitigation of outbreaks [5]. Strengthening international collaboration is also critical given the transboundary nature of zoonotic disease emergence and the increasing risks associated with globalization and climate change.

In conclusion, hantavirus infections represent a paradigm of environmentally driven emerging zoonoses. Their expanding epidemiology, the emergence of travel-associated exposure events, and the unique epidemic potential of the Andes virus collectively highlight the urgent need for enhanced surveillance, interdisciplinary research, and global health preparedness. Strengthening One Health approaches and investing in vaccine and antiviral development will be essential to mitigating the future burden of hantavirus disease.

## CRediT authorship contribution statement

**Christian Joseph N. Ong:** Writing – original draft, Formal analysis, Data curation, Conceptualization. **Kevin Smith P. Cabuhat:** Writing – review & editing, Writing – original draft, Formal analysis, Conceptualization. **Jamil Allen G. Fortaleza:** Writing – review & editing, Validation, Investigation. **Rich Milton R. Dulay:** Writing – review & editing, Validation, Supervision.

## Ethical approval and consent to participate

Not applicable.

## Availability of data

Not applicable.

## Consent for publication

Not applicable.

## Funding

None.

## Declaration of competing interest

The authors declare no competing interests that could have influenced the content of this letter. The authors declare they have no known competing financial interests or personal relationships that could have appeared to influence the work reported in this paper.
